# Potential of an Attractive High-Rate Navel Orangeworm (Lepidoptera: Pyralidae) Pheromone Dispenser for Mating Disruption or for Monitoring

**DOI:** 10.3390/insects15110884

**Published:** 2024-11-12

**Authors:** Charles S. Burks, Bradley S. Higbee

**Affiliations:** 1 USDA, Agricultural Research Service, San Joaquin Valley Agricultural Sciences Center, 9611 South Riverbend Avenue, Parlier, CA 93648, USA; 2 Trécé Inc., Adair, OK 74330, USA; buglimo@gmail.com

**Keywords:** navel orangeworm, *Amyelois transitella*, monitoring, mating disruption, sex pheromone, almond, pistachio

## Abstract

Mating disruption is used to manage *Amyelois transitella* (Walker) (Lepidoptera: Pyralidae). Current mating disruption uses a single compound, but at least one other is necessary for source contact in laboratory assays and efficient capture in traps in the field. We conducted experiments to examine disruption of sexual communication and attraction of males to these high-rate dispensers with either one or both compounds. Trap suppression experiments compared males captured in pheromone traps in orchard blocks treated at a low, medium, or high number of dispensers per ha, and a trapping study used the dispensers as attractants in sticky traps to compare their relative attractiveness. The 2-compound dispensers suppressed male capture in pheromone traps more effectively than the 1-compound dispensers at the intermediate density. The trapping study found that both dispensers captured fewer males than a monitoring lure in the absence of mating disruption but more males in the presence of mating disruption. These data provide additional support for a hybrid mechanism of mating disruption for this species and indicate that the use of the more attractive 2-compound dispensers could make mating disruption more effective at the lowest dispenser density currently used. Alternatively, a lure base on the 2-compound dispenser could provide equally effective monitoring in the presence of mating disruption.

## Simple Summary

The navel orangeworm is an important pest of high-value crops including almonds, pistachios, and walnuts. Monitoring and mating disruption are important tools for control of this moth, and improved understanding of its response to pheromone composition and concentration has the potential to improve monitoring and mating disruption. Experiments with high-release rate passive diffusion pheromone dispensers revealed that capture of males in pheromone traps was suppressed more successfully at an intermediate rate of dispensers per acre when a second pheromone component was included. Traps baited with part or all of the single compound dispensers as bait were minimally attractive in the absence of mating disruption, thereby confirming earlier research on the mechanism of mating disruption for navel orangeworm. Traps baited with the two-compound dispensers captured similar numbers of navel orangeworm in either the presence or absence of mating disruption, indicating that this dispenser could also be used to improve monitoring. This study provides scientists and extension personnel with further insight into the mechanism of the current widely used mating disruption products for navel orangeworm and indicates that the two-compound dispensers could provide improvements in monitoring for navel orangeworm compared with the presently-used products.

## 1. Introduction

Lepidopteran sex pheromones are used in a variety of pest management tactics [1,2,3]. The most widely used applications are monitoring [4] and mating disruption [5,6,7], although other uses include mass-trapping [8,9,10,11,12], attract-and-kill [13,14,15,16,17], and push–pull strategies [18,19].

Mating disruption works by preventing or delaying mating, but how this is accomplished varies with the neurophysiology of the target species and the formulation used [5]. A central distinction is whether interaction of the target pest with the pheromone dispenser is required for disruption (competitive mating disruption) or if instead the formulation can prevent males from mating with females without retaining them at the dispenser (non-competitive mating disruption). A third intermediate was proposed, in which orientation to dispensers is initially involved but males are then desensitized and the disruption is non-competitive in nature [5]. For the codling moth *Cydia pomonella* (Linnaeus) (Lepidoptera: Tortricidae), a very well-studied example of competitive mating disruption [5], males remain capable of orienting to sex pheromone from a calling female even after exposure to a very high pheromone concentration such as that from an aerosol dispenser [20,21]. In contrast, males of the oriental fruit moth *Grapholita molesta* (Busck) (Lepidoptera: Tortricidae), a well-documented example of non-competitive mating disruption, become desensitized and do not orient to either dispensers or females when they encounter pheromone from dispensers with a release rate high enough to exceed a critical threshold [5,22]. Non-competitive mating disruption is thought to be effective at higher target pest populations compared with mating disruption by a competitive mechanism [5]. Habituation or desensitization is an important aspect of non-competitive mating disruption, and it has been theorized that mating disruption has been developed more thoroughly in Lepidoptera in part because such reactions are more common in Lepidoptera than other orders [23]. In some moths, both the lower and upper thresholds are increased in response to exposure to supranatural sex pheromone concentrations [24].

Common methods to apply mating disruption in orchards include high-density passive dispensers, used at a rate of 300–1000 per ha; low-density passive dispensers, used at a rate of around 40–70 per ha; and aerosol dispensers, used at a rate of 2–5 per ha [5,7] (see Section 4.1).

Advantages of sex pheromone for monitoring, including efficiency of attraction and species specificity, are generally lost if pheromone is used for mating disruption. Initially lures with a higher emission rate were used to monitor codling moth in the presence of mating disruption [25]. More recently, however, lures based on host volatiles and other natural products have been developed for monitoring codling moth and other lepidopteran pests in the presence of mating disruption [26,27,28,29,30,31,32]. Capturing non-target species (by-catch) can complicate the use of such compounds [33]. Lights from small light-emitting diodes have been used to enhance response to these products [34].

The navel orangeworm *Amyelois transitella* (Walker) (Lepidoptera: Pyralidae) is a key pest of almond, pistachio, and walnut in California [35]. Mating disruption is an established practice for this species [36,37,38]. Current formulations include aerosol products from several manufacturers and a low-density passive diffusion dispenser product [38]. The pheromone blend of the navel orangeworm is unusual because it contains both type I straight-chain compounds and type II polyunsaturated long-chain hydrocarbons [39,40]. An effective pheromone monitoring lure is available using both the type I and type II compounds [41,42,43], but the mating disruption products use only a single compound, (11Z,13Z)-hexadecadienal (Z11,Z13-16Ald), because of regulatory and economic factors favoring mating disruption with the single-compound product [44]. The mechanism is thought to be hybrid, based in part on analysis using capture as a function of dispenser density [37]. Wide-spread use of mating disruption complicates the use of pheromone lures for monitoring [43], including the use of ground nuts to trap eggs or adult females [45,46], and a natural product (phenyl propionate) used by itself or synergized with a pheromone monitoring lure [47].

In this study, we compare the existing low-density passive dispenser containing only Z11,Z13-16Ald (hereafter referred to as a single-compound dispenser) to mating disruption and monitoring with low-density passive dispensers containing Z11,Z13-16Ald and the type II compound (3Z,6Z,9Z,12Z,15Z)-tricosapentaene (TCP) (hereafter referred to as a two-compound dispenser). Mating disruption and trapping experiments were conducted to explore the practical use of a more attractive dispenser. A trial comparing the ability of these two dispensers to suppress pheromone traps was conducted in almonds at the end of the season due to the necessity to conduct the trial under a crop-destruct protocol. In subsequent experiments, the attractiveness of the dispensers was compared by using part or all of the dispensers as lures in traps in the absence or in the presence of mating disruption for navel orangeworm.

## 2. Materials and Methods

### 2.1. Mating Disruption Products

The mating disruption devices tested were black polyvinyl chloride dispensers providing a total of 22 g active ingredient (a.i.) per acre when placed at a density of 28 dispensers per acre. The a.i. consisted of either a single-compound formulation containing Z11,Z13-16Ald alone (TRE 1451) or a two-compound blend containing a 19:1 ratio of Z11,Z13-16Ald and TCP (TRE 1450) (hereafter 1-compound and 2-compound, respectively). There are two additional pheromone components, (Z11,Z13)-hexadecadien-1-ol and (Z11,Z13)- hexadecadien-1-ol, that are necessary and sufficient for an optimally attractive navel orangeworm sex pheromone blend, but the two compounds used in TRE 1450 are sufficient to attract males to a point source [40].

### 2.2. Effect of Dispenser Blend and Density on Trap Suppresion

**Figure 1:**
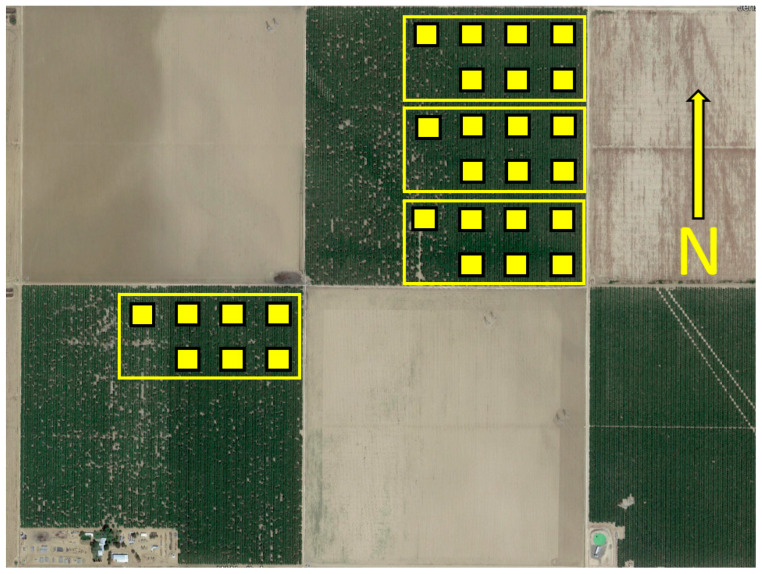
Plot arrangement for the trap suppression experiment. Four replicate blocks contained seven plots each. The untreated control plot was in the isolated position to the west in all cases. The six levels of blend formulation and dispenser density were randomized among the eastern six positions. The fields containing the replicate blocks were approximately 800 × 800 m.

A randomized complete block design (RCBD) was used to examine the effects of blend and dispenser density in a series of 0.2 ha plots in approximately 25-year-old almond orchards in September and early October of 2016 (Figure 1). These orchards near Firebaugh CA (36.70, 120.63) were planted with 6.7 m row spacing and 5.5 m between trees within rows. Because of more stringent regulatory requirements for tests with TCP, this study was conducted in two adjacent 24 ha almond orchards scheduled for removal prior to the next growing season and then only after the final commercial harvest. The eastern half of each of these orchards was used because there were more missing trees on the west half. Three densities were used for each of the two formulations for a total of six treatments. These six treatments were assigned randomly to each of four replicate blocks.

The mating disruption treatment was applied at three dispenser densities: 69, 30, and 17 dispensers per ha (respectively 53, 24, and 14 g a.i. per ha). These densities respectively corresponded to every second, third, and fourth tree. The two higher densities are similar to the range of 38 to 70 dispensers/ha in the label of CIDETRAK NOW MESO dispensers (Trece Inc., Adair, OK, USA). There was a buffer between the treatment plots of ≥44 m in the north–south (in-row) direction and ≥54 m in the east–west (cross-row) direction. The effect of mating disruption on navel orangeworm response to pheromone was assessed using white wing traps (PHEROCON 1C, Trécé Inc., Adair, OK, USA) baited with commercial pheromone lures (PHEROCON NOW L2-H, Trece Inc., Adair, OK, USA). Four pheromone traps per plot were used, placed as nearly as possible in the middle between dispensers. Multiple traps per plot were used only to improve detection (i.e., increase the number of males captured), and plot sums were used as response variables in analyzing the results. Control plots, in which there was no mating disruption, were located to the west (i.e., upwind) of the northern three plots per replicate block, at a distance corresponding to the separation of monitoring traps in the other plots. The pheromone sources used were a commercial pheromone lure (PHEROCON NOW L2-H).

This experiment was conducted at three monitoring intervals. In the first interval, from 21 September to 28 September, the monitoring traps were baited with pheromone monitoring lures as described above. In the second interval, from 28 September to 5 October, sticky liners were changed and traps were baited with unmated females in the same manner as a previous experiment with attractive blends for navel orangeworm [44]. Briefly, females were isolated as larvae, allowed to emerge as adults, and three unmated females were sealed in a mesh bag and hung in a sticky trap in place of a pheromone lure. For details, see Burks and Brandl [48]. In the third interval, from October 5 to 13, the sticky liners were again removed and the mesh bags with unmated females were removed and replaced with pheromone monitoring lures.

An additional experiment, conducted between 7 October and 13 of 2016, compared the attractiveness of the 2-compound and 1-compound blends when used as attractants in traps. An RCBD experiment with eight replicates compared males captured in three treatments: (1) wing traps with a navel orangeworm pheromone monitoring lure (NOW Biolure, Suterra LLC, Bend, OR, USA); (2) wing traps with a 2.5 cm segment of the 2-compound blend (intact dispenser 21.6 cm); or (3) wing traps with a 2.5 cm segment of 1-compound formulation (intact dispenser 30.5 cm). In all cases, lures or putative attractants were hung from the upper surface of the wing trap. This experiment was conducted in a mature 16 ha almond orchard near Parlier (36.65°, 119.52°). The orchard was planted with the varieties “Nonpariel” and “Monterey” in 6.7 m rows running east to west, with 4.9 m spacing between trees within rows. Orchard rows served as replicate blocks, with treatments randomly assigned to one of three positions within these replicates. Traps were placed ≥ 27 m from each other and from the edge of the orchard.

### 2.3. Attraction of Males to Dispensers in Sticky Traps

In 2019, two RCBD experiments further examined whether NOW males were captured in traps containing segments of 1- or 2-compound dispensers. For each of the dispenser types, portions of dispensers cut to one of two sizes were compared with an entire dispenser cut into sections to fit inside a sticky trap. Treatments were sections of either the 21.6 cm 1-compound dispenser or the 30.5 cm 2-compound dispenser. Segments of either 2.5 cm, 10.2 cm (1/3 of a 2-compount dispenser), or 10.8 cm (1/2 of a 1-compound dispenser) were hung inside an orange Wing Trap (Suterra, Bend, OR, USA) from a wire hook fabricated in the middle. Wing traps were placed in bent-wire frames per standard practice at USDA-ARS Parlier [49]. Pieces cut to approximately 4 inches to facilitate hanging them from the hook from a 0.15 cm hole drilled close to one edge in the center of the MESO (to hang pieces horizontally). The upper “dose” was two segments of the 1-compound lure or three segments of the 2-compound lure to simulate getting the entire dispenser inside the trap.

The first experiment, conducted in the absence of mating disruption, compared the number of navel orangeworm adults captured in traps baited within a mature pistachio orchard (cv “Kerman”) planted in 5.5 m rows with 4.9 m between trees within rows. In this experiment, the six dispenser treatments described above were compared with a blank wing trap (no attractant, negative control) and a wing trap baited with a standard pheromone monitoring lure as described in the previous section (positive control). This first experiment was conducted over 4 weeks, from 2 August to 27 August.

A subsequent experiment was continued at a mature almond orchard under mating disruption provided by aerosol dispensers (Isomate NOW Mist, Pacific Biocontrol, Vancouver, WA, USA). For this experiment, the negative control was a pheromone monitoring lure, since previous studies showed that, under mating disruption, capture in traps baited with these lures is completely suppressed (Burks et al., 2020, [47]). Wing traps baited with a commercial lure emitting PPO (PHERECON-HR L2, Trece Inc., Adair, OK, USA) synergized by a separate pheromone lure (NOW Biolure, Suterra LLC, Bend, OR, USA) served as a positive control (Burks et al., 2020, [47]). This experiment was conducted in a mature almond orchard planted in alternating rows of “Nonpareil” and “Monterey”, with 7.3 m between rows and 6.7 m between trees within rows. Rows used as replicate blocks were approximately 50 m apart, and trap positions within replicated blocks were approximately 50 m apart. This second experiment was conducted from 28 August to 10 September.

### 2.4. Data Analysis

Data summary and analysis were performed using R 4.4.1 [50], using the packages lme4 [51], car [52], and emmeans (Lenth 2024) [53]. For the 2016 trap suppression experiments, male capture was compared between control traps with no mating disruption vs. all dispenser formulations and densities using the Kruskal–Wallis rank sum test, stats::kruskal.test(). Trap capture was compared between formulations and densities (excluding the untreated control plots) using a generalized linear mixed model (GLMM) with a negative binomial distribution of error using lme4::glmer.nb(). Initially, the analysis was performed as a factorial combination of formulation*density. Based on a significant interaction for the first monitoring interval, density was then compared separately for each of the two formulations. This approach was also taken for the third monitoring interval for consistency. An ANOVA-style analysis of deviance was conducted using car::Anova(), and a Tukey means separation test was conducted using emmeans::pairs(). Differences in trap captures were not analyzed for period 2 (traps baited with unmated females) because too few males were captured to make statistical comparisons. Attraction in pheromone traps in 2016 was compared between treatments by examining the 95% confidence interval of the mean [t.test(), univariate] for treatments with capture > 0. For the 2019 data, trap capture was compared between attractants using a 1-way ANOVA as described above.

## 3. Results

### 3.1. Effect of Dispenser Blend and Density on Trap Suppresion

Comparison of all mating disruption treatments to untreated controls revealed significantly fewer males in traps in period 1 (χ^2^ = 9.95, df = 1; *p* = 0.0016), period 2 (χ^2^ = 17.17, df = 1; *p* < 0.001), and period 3 (χ^2^ = 10.06, df = 1; *p* = 0.0015) (Table 1). An initial factorial analysis of males per plot in plots treated with dispensers in period 1 revealed a significant interaction between formulation and dispenser density (χ^2^ = 8.90, df = 2; *p* = 0.0117). Trap capture was examined as a function of density separately for plots treated with 1-compound and 2-compound dispensers.

There were significant differences in trap capture among plots treated with the 1-compound dispenser formulation in period 1 (χ2 = 48.76, df = 2; *p* < 0.001). Trap capture in the intermediate (30 dispensers/ha) density was not significantly different from trap capture in the low-density plots (17 dispensers/ha), while the plots treated with the highest density (69 dispensers/ha) had significantly fewer males captured per plot compared with the two lower densities (Table 2). In contrast, in plots treated with the 2-compound dispenser formulation in period 1, trap capture was significantly different in all three dispenser densities (χ2 = 76.66, df = 2; *p* < 0.001) (Table 2).

Similar numerical trends were seen in period 3 (Table 3), although fewer males were captured at all dispenser densities. In plots treated with 1-compound dispensers, there were no significant differences in males captured among the three dispenser density levels (χ2 = 3.19, df = 2; *p* = 0.203) (Table 3). There were, however, significant differences in trap capture between plots treated with different dispenser densities for the 2-compound formulation for period 3 (χ2 = 16.43, df = 2; *p* = 0.0002). Plots with the highest density of 2-compound dispensers had significantly lower trap capture than plots with the lowest density, whereas the plots at the intermediate density had intermediate trap capture (Table 3).

In the 2016 comparison of attractants, no males were captured in traps baited with a 2.5 cm segment of the 1-compound formulation (Table 4). For traps baited with a 2.5 cm segment of the 2-compound formulation or with a commercial pheromone monitoring lure, the lower 95% confidence limits were >0 in both cases, while the 95% confidence intervals overlapped between these two treatments (Table 4).

### 3.2. Attraction of Males to Dispensers in Sticky Traps

In the absence of mating disruption, there were significant differences among attractants tested in sticky traps (χ2 = 513.67, df = 7; *p* < 0.0001) (Table 4). When partial or whole dispensers were compared, blank traps or traps baited with a commercial pheromone lure, the attractant types (blank trap, 1-compound dispenser, 2-compound dispenser, or monitoring lure) were generally in different ranges in the multiple-range Tukey test (Table 5). The 1-compound dispenser treatments all captured ≥20 × the number of NOW captured in a blank trap, and all were different at a significance level of *p* < 0.01, but only the 10.8 cm 1-compound segments were significantly different at a level of *p* < 0.05 (Table 5). In general, the level of trap capture in the absence of mating disruption was monitoring lure > 2-compound dispenser segments > 1-compound dispenser segments > blank trap.

There were also significant differences among attractants tested in sticky traps in the presence of mating disruption (χ2 = 146.67, df = 7; *p* < 0.0001). In this experiment, there was no significant difference in trap capture between the commercial lure used for trapping in the presence of mating disruption (traps with a PPO lure and pheromone lure) and the 2-compound dispenser at any of the segment sizes tested (Table 6). Traps baited with 2-compound dispenser segments of any size captured more adults than traps baited with 1-compound dispenser segments of any size. There were, however, differences in the number of adults captured between traps baited with 1-compound dispenser segments of different sizes. For the 1-compound formulation, traps baited with the entire dispenser a 10.8 cm segment captured significantly more adults than traps baited with pheromone monitoring lures alone, whereas traps baited with a 2.5 cm segment did not capture significantly more adults than traps baited with a pheromone lure.

## 4. Discussion

The experiments reported here examine both trap suppression and mating using 2-compound high emission rate dispensers. The initial interest in comparing attraction between 2-compound high emission rate dispensers and alternative attractants was to inform interpretation of the trap suppression studies. However, given economic barriers to commercial production of mating disruption products containing a type II pheromone, applications to monitoring might be a more practical use of these dispensers (discussed below).

### 4.1. Categories of Mating Disruption Dispensers

A previous review [5] described categories of mating disruption dispensers that included, among others, high-density passive dispensers, used at high densities that usually equated to one or more dispensers per tree; intermediate-density passive dispensers, used at densities equivalent to one device every several trees and rows; and aerosol dispensers, placed more sparsely by about an order of magnitude. In the previous review [5], these were referred to respectively as “hand-applied dispensers”, “meso dispensers”, and “mega dispensers”. We believe that the terms used here (high-density passive, low-density passive, and aerosol) are more appropriate because “meso dispenser” is too similar to the claimed trademark MESO^TM^, and currently aerosol dispensers are the only example of the mega category. In the case of the navel orangeworm, both low-density passive dispensers and aerosol dispensers are efficacious for protection of almonds from navel orangeworm damage [38], so the choice of which system to use is guided by user evaluation of price, ease of application, and mechanical reliability. 

### 4.2. Mechanism of Trap Suppression as Informed by the Attraction Experiments

Interpretation of the data of the mating disruption (trap suppression) data reported here is aided by the tests of the 1- and 2-compound dispensers as attractants. The finding that traps baited with 1-compound dispensers in a field without mating disruption captured marginally more males than blank traps is consistent with past laboratory and field experiments [40,54,55]. In contrast, the traps baited with two-compound dispensers captured 30 to 45% of the males captured in an optimized pheromone monitoring lure in the absence of mating disruption. In the presence of mating disruption, traps baited with pheromone monitoring lures captured negligible numbers of males, similar to the number of moths in blank traps in the trapping experiment in the absence of mating disruption. As in the experiment without mating disruption, traps baited with 1-compound dispensers in the presence of mating disruption captured numerically more males than blank traps and were generally in a different range in the Tukey multiple range test. Traps baited with 2-compound dispensers in the presence of mating disruption captured 30 to 90% of the most effective treatment, PPO with a pheromone monitoring lure.

These observations can be used to infer information about the impact of mating disruption on the relative attractiveness of female-strength monitoring lures and the dispensers. Data from previous studies indicate that capture in traps baited with pheromone monitoring lures in the presence of mating disruption is similar to capture in blank traps in the absence of mating disruption [56], that traps baited with unmated females and traps baited with monitoring lures capture similar numbers of males with monitoring intervals of ≥3 days [42], and that capture in traps baited with pheromone monitoring lures alone and traps baited with PPO and a monitoring lure are similar when far from mating disruption influence [43]. Thus, these data indicate that the low capture in traps baited with 1-compound dispensers and the intermediate capture in traps baited with 2-compound dispensers were similar between a non-mating disruption orchard in which pheromone monitoring lures were highly effective and a mating disruption orchard in which pheromone lures were completely suppressed. This increase in both the lower and the upper response threshold is similar to observations with other Phycitinae such as the almond moth *Cadra cautella* (Walker) [24] and the Indian meal moth *Plodia interpunctella* (Hübner) [57].

The evidence that both the 1-compound and 2-compound dispensers are attractive to a degree is consistent with the hybrid mechanism of mating disruption for navel orangeworm [37], previously determined by graphical analysis of trap capture as a function of dispenser density [5]. In graphs of such data, a hybrid mechanism shows characteristics similar to a competitive mechanism at low dispenser density but has characteristics of a non-competitive mechanism at higher densities. This is thought to be because males are attracted to dispensers from a distance but become desensitized to pheromone at closer distances and higher pheromone concentrations [5]. That previous analysis of mechanism for navel orangeworm was conducted with aerosol dispensers [37]. The resources available in the current study did not allow a formal analysis of capture vs. dispenser density for dispensers, but these data are consistent with and further support the characterization of mating disruption for navel orangeworm as a hybrid mechanism.

The data from the trap suppression experiments indicate that the 2-compenent dispensers were more effective at the intermediate dispenser density of 30 dispensers per ha, as indicated by the different multiple range groupings for the intermediate density between the two formulations. This suggests that treatment with the more attractive 2-compound blend would be particularly likely to be more efficacious at the lower end of the dispenser density recommended by the label, which some users might view as an advantage.

The findings from the trap suppression experiment are generally consistent with those from a previous season-long study of mating disruption with either Z11,Z13-16Ald alone or two versions of a more complete blend in aerosol dispensers [44]. The second interval in the current study, using unmated females, was conducted for compatibility with the previous study in which assays were conducted with unmated females but not pheromone monitoring lures (which were then unavailable). Trap suppression [100 × 1 − mating disruption/comparison] was over 99% in almonds in that study and nominally 77 to 99% in pistachios. The suppression index in pistachios, however, was probably curtailed by the reduction of trap efficiency in control blocks due to trap saturation due to captures of >150 males/trap [58]. In that study, there was no significant difference between the 1-compound and more complete formulations in males per trap in either crop, although numerical trends in pistachios suggested that the more complete blends reduced trap capture more effectively. In that study the only statistically significant effect was in prevention of mating in sentinel females (not used in the current study), for which both of the more complete blends were in a different multiple range from the 1-compound blend, which in turn had significantly less mating than the untreated control. In the present experiment, the overall suppression indexes for all treatments (Table 1) are 88.5, 99.7, and 97.6% for the three intervals. The trap suppression for female-baited traps in this study is thus similar to suppression in almonds in the earlier study. The daily high and low temperatures were seasonal and similar during the three intervals. In the present study, the experiments using pheromone monitoring lures provided more statistically robust comparisons compared with current or previous studies using unmated females. We are unaware of rigorous tests for pheromone autodetection [59] by females in navel orangeworm, so it is unknown whether that might contribute to differences observed between experiments using traps baited with females and those conducted using artificial pheromone lures.

### 4.3. Practical Application of the 2-Compound High Rate Dispenser

Ideally, the trap suppression study reported here would be followed up by a season-long replicated study of larger plots to compare crop damage between mating disruption treatments and a control. However, under current U.S. regulation, commercialization of a pheromone blend containing a type II compound (required for attractiveness for navel orangeworm) would require more extensive and expensive testing compared with the current formulations based on type I straight-chain Lepidopteran pheromones, which are categorized as “Generally Regarded as Safe” [44]. These impediments to commercialization were also identified in a previous study of aerosol mating disruption using more attractive pheromone blends for the navel orangeworm [44].

The 2-compound dispenser could nonetheless serve a useful role in monitoring for navel orangeworm. The data from this study indicates that these dispensers capture males in both the presence and absence of aerosol mating disruption. Traps baited with the 2-compound dispensers do not capture as many males as traps baited with PPO and a pheromone monitoring lure, but there is also less problem with capture of non-target insects (by-capture). However, because of differences in registration for monitoring products compared with products for mating disruption, these impediments are not as applicable for use of the 2-compound dispensers for a monitoring product.

## 5. Conclusions

Experiments with traps baited with dispensers containing either (11Z,13Z)-hexadecadienal alone or in a 19:1 ratio with (3Z,6Z,9Z,12Z,15Z)-tricosapentaene found the aldehyde-only dispensers marginally attractive and the aldehyde-plus-hydrocarbon dispensers much more attractive. This observation adds to evidence for a hybrid mechanism of mating disruption for navel orangeworm. Trap suppression studies indicated that the two-compound dispensers were more effective at reducing trap capture at the lower end of the range of density (dispensers/ha) found in the current product label, suggesting that this 2-compound product could have a practical advantage for use in mating disruption. The observation of similar moderate levels of trap capture with low by-capture also suggests that a product based on the 2-compound dispenser could also be useful for monitoring for navel orangeworm.

## Figures and Tables

**Table 1:** Males per plot (mean ± SEM, n = 4) in plots in untreated plots and plots treated with high-rate passive dispensers.

Period	No Mating Disruption	Mating Disruption ^1^
21 September to 28	260 ± 80	30 ± 6.5 **
28 September to 5 October	63 ± 31	0.2 ± 0.13 ***
5 October to 13 October	248 ± 73	6 ± 1.3 **

^1^ Mean for all mating disruption treatments. Kruskal–Wallis rank sum test by row, ** *p* < 0.01; *** *p* < 0.001.

**Table 2:** Males per plot (mean ± SEM, n = 4) in plots treated with high-rate passive dispensers containing one or two pheromone components, at 17, 30, or 69 dispensers per ha, 21 September to 28 (period 1).

Dispensers/ha	Single Compound Dispensers	Two Compound Dispensers ^1^
17	57 ± 15.7 a	63 ± 16 a
30	37 ± 16.7 a	15 ± 3.6 b
69	7 ± 2.8 b	3 ± 0.8 c

^1^ Means in the same column followed by different letters are significantly different (GLMM with nb distribution, experiment-wise *p* < 0.05).

**Table 3:** Males per plot (mean ± SEM, n = 4) in plots treated with high-rate passive dispensers containing one or two pheromone components at 17, 30, or 69 dispensers per ha, 5–13 October (period 3).

Dispensers/ha	Single Compound Dispensers	Two Compound Dispensers ^1^
17	8.8 ± 4.37 a	12 ± 3.3 a
30	5 ± 3.7 a	3.8 ± 1.7 ab
69	2 ± 2.3 a	1 ± 0.71 b

^1^ Means in the same column followed by different letters are significantly different (GLMM with nb distribution, experiment-wise *p* < 0.05).

**Table 4:** Males per trap (mean, 95% CI in parentheses, n = 8) for dispensers and monitoring lures in the absence of mating disruption, October 2016.

Attractant	Adults per Trap
Single compound dispenser, 2.5 cm segment	0
Two compound dispenser, 2.5 cm segment	2.9 (1.18, 4.57)
Pheromone monitoring lure	7.0 (2.58, 11.42)

**Table 5:** Adults per trap per week (mean ± SEM, n = 8) for dispensers and monitoring lures in the absence of mating disruption, August 2019.

Attractant	Adults per Trap ^1^
Blank trap	0.03 ± 0.03 a
Single compound dispenser, 2.5 cm segment	0.8 ± 0.34 ab
Single compound dispenser, 10.8 cm segment	0.9 ± 0.19 b
Single compound dispenser, entire	0.7 ± 0.17 ab
Two compound dispenser, 2.5 cm segment	23 ± 6.6 c
Two compound dispenser, 10.2 cm segment	22 ± 3.9 c
Two compound dispenser, entire	32 ± 5.3 c
Pheromone monitoring lure	71 ± 7.9 d

^1^ Means followed by different letters are significantly different (GLMM with negative binomial, experiment-wise *p* < 0.05).

**Table 6:** Adults per trap per week (mean ± SEM, n = 8) for dispensers and monitoring lures in the presence of mating disruption, August 2019.

Attractant	Adults per Trap ^1^
Pheromone monitoring lure	0.06 ± 0.06 a
Single compound dispenser, 2.5 cm segment	0.25 ± 0.095 ab
Single compound dispenser, 10.8 cm segment	1.8 ± 0.62 c
Single compound dispenser, entire	1.5 ± 0.37 bc
Two compound dispenser, 2.5 cm segment	8 ± 3.0 d
Two compound dispenser, 10.2 cm segment	24 ± 5.0 d
Two compound dispenser, entire	14 ± 3.6 d
Pheromone monitoring lure with PPO dispenser	25 ± 3.5 d

^1^ Means followed by different letters are significantly different (GLMM with negative binomial, experiment-wise *p* < 0.05).

## Data Availability

Data and scripts for supporting this study are in a Zenodo repository, doi:10.5281/zenodo.14027049.
